# Cannabidiol sensitizes triple-negative breast cancer cells to NK cell-mediated killing via EGFR inhibition and FAS upregulation

**DOI:** 10.1186/s42238-025-00340-5

**Published:** 2025-11-04

**Authors:** Perawat Garunyapakun, Boonyanuch Ramwarungkura, Krissada Natungnuy, Chairat Turbpaiboon, Pa-thai Yenchitsomanus, Mutita Junking

**Affiliations:** 1 Department of Anatomy, Faculty of Medicine Siriraj Hospital, Mahidol University, Bangkok, 10700 Thailand; 2 Department of Medical Science, Faculty of Science, Rangsit University, Pathumthani, 11110 Thailand; 3 Division of Molecular Medicine, Research Department, Faculty of Medicine Siriraj Hospital, Mahidol University, Bangkok, 10700 Thailand; 4 Siriraj Center of Research Excellence for Cancer Immunotherapy (SiCORE-CIT) and Division of Molecular Medicine, Research Department, Faculty of Medicine Siriraj Hospital, Mahidol University, Bangkok, 10700 Thailand; 5 Graduate Program in Biomedical Sciences, Faculty of Medicine Siriraj Hospital, Mahidol University, Bangkok, 10700 Thailand

**Keywords:** Cannabidiol, Triple-negative breast cancer, FAS, EGF, EGFR

## Abstract

**Background:**

Triple-negative breast cancer (TNBC) is a highly aggressive subtype lacking targeted therapies, presenting a significant clinical challenge. The epidermal growth factor receptor (EGFR) plays a crucial role in TNBC progression, making it a promising target for therapeutic intervention. This study investigated the potential of cannabidiol (CBD) as a therapeutic agent that targets EGFR and associated signaling pathways in TNBC.

**Methods:**

The TNBC cell lines MDA-MB-468 and MDA-MB-231 were treated with CBD in the presence or absence of epidermal growth factor (EGF). Cell proliferation, FAS protein expression, and activation of the EGFR signaling pathway were assessed. The cytotoxic effects of CBD on TNBC cells and natural killer (NK) cells were also evaluated.

**Results:**

CBD significantly elevated FAS protein expression in MDA-MB-468 cells compared to EGF treatment alone (125.29 ± 5.87% vs. 83.07 ± 1.30%, *p* < 0.0001). Further molecular analysis revealed that CBD inhibited EGFR signaling by downregulating key oncogenic proteins, including KRAS, PI3K, and AKT. Moreover, CBD enhanced the cytotoxic effects of NK-92 cells, reducing the viability of MDA-MB-468 cells more effectively than EGF alone did (52.12 ± 1.28% vs. 113.69 ± 1.68%, *p* < 0.0001).

**Conclusions:**

These findings suggest that CBD holds promise as a potential anticancer agent in TNBC by disrupting EGFR signaling and promoting apoptosis. However, further studies are necessary to optimize its therapeutic window and minimize adverse effects, particularly regarding its potential cytotoxicity to immune cells.

**Supplementary Information:**

The online version contains supplementary material available at 10.1186/s42238-025-00340-5.

## Introduction

Triple-negative breast cancer (TNBC) is a subtype of breast cancer characterized by the absence of estrogen receptor (ER), progesterone receptor (PR), and human epidermal growth factor 2 (HER2) receptors. TNBC is particularly aggressive and is associated with relatively high rates of recurrence and mortality [1–3]. While targeted therapies for TNBC are being developed, they remain largely inaccessible [4]. The epidermal growth factor receptor (EGFR) is commonly overexpressed in TNBC and is associated with poor prognosis and increased recurrence rates [5, 6]. EGFR overexpression in TNBC is believed to drive cancer progression through multiple mechanisms, including increased cell proliferation, migration, invasion, angiogenesis [7, 8], and the inhibition of apoptosis [9]. In both TNBC cell lines and animal models, EGFR inhibitors have shown promise in limiting tumor growth, and several EGFR inhibitors are currently undergoing clinical trials for the treatment [10, 11]of TNBC.

The EGF/EGFR signaling pathway may play a role in regulating FAS-mediated apoptosis in cancer cells. Blocking EGFR signaling could increase susceptibility to FAS-mediated apoptosis, making it a promising therapeutic target in cancer [12]. However, the specific mechanism by which EGFR influences FAS-mediated apoptosis is not yet fully understood, particularly with respect to the ligands, such as TNF and IL-2, that activate the FAS receptor. Immune cells, including natural killer (NK) cells, release cytotoxic ligands that bind to receptors on target cells and induce cell death. NK cells, key components of the innate immune system, exhibit strong antitumor activity by releasing a variety of cytotoxic cytokines [13]. One such mechanism involves the FAS ligand, a cytokine that binds to the FAS receptor on cancer cells [14], triggering an apoptotic cascade within the tumor cell and leading to its destruction. These findings highlight the crucial role of NK cells and their cytokines in the defense of the immune system against malignancies. Additionally, cancer cells can downregulate FAS expression or alter the *FAS* gene, impairing its function [15]. Mutations, such as KRAS G12D, can also reduce FAS production, enabling cancer cells to evade immune surveillance and increase survival [11]. As a result, targeting the FAS pathway is emerging as a promising therapeutic strategy for cancer treatment.

Phytochemicals have demonstrated anticancer properties in breast cancer, including inhibition of tumor growth [16], induction of apoptosis [17], and enhancement of the immune response [18]. Notable phytochemicals with potential for breast cancer treatment include curcumin [19], resveratrol [20], flavonoids [21], and indole-3-carbinol [22], which have been shown to be effective in preclinical studies. Cannabidiol (CBD), a compound derived from the cannabis plant, has gained attention as a promising anticancer agent [23]. CBD has exhibited anticancer effects [24, 25], such as inhibiting cell proliferation, inducing apoptosis, suppressing angiogenesis, and modulating the immune response [26, 27]. In TNBC, the potential therapeutic role of CBD may involve inhibiting proliferation via the EGF/EGFR pathway and promoting apoptosis through signaling interactions [7–9, 28]. This study investigated EGFR expression in TNBC cell lines and examined its effects on cell proliferation and FAS expression.

Our findings suggest that CBD can suppress TNBC cell proliferation in the presence of EGF while simultaneously increasing FAS protein expression. This dual action indicates that CBD inhibits proliferation and enhances FAS-mediated apoptosis in TNBC cells, increasing their vulnerability to NK-92 cell-mediated cytotoxicity by activating apoptotic proteins. This study highlights the potential of CBD as a therapeutic agent in TNBC, offering promising insights for further research and the development of novel treatments for TNBC.

## Materials and methods

### Cell cultures

Breast cancer cell lines (MDA-MB-468, MDA-MB-231, and MCF-7) and the human natural killer (NK) cell line NK-92 were obtained from the American Type Culture Collection (ATCC; Manassas, VA, USA). The breast cancer cell lines were cultured in Dulbecco’s modified Eagle’s medium (DMEM; Gibco, Invitrogen, Carlsbad, CA) supplemented with 10% fetal bovine serum (FBS; Gibco), 100 U/ml penicillin, and 0.1 mg/mL streptomycin. NK-92 cells were cultured in Minimum Essential Medium Eagle (MEM) supplemented with 2 mM L-glutamine, 0.02 mM inositol, 0.1 mM 2-mercaptoethanol, 0.02 mM folic acid, 100–200 U/mL recombinant IL-2 (R & D system, Minneapolis, MN, USA), and 12.5% fetal bovine serum. All the cell cultures were maintained at 37 °C in a humidified incubator with 5% CO_2_.

### Flow cytometry

The surface expression of EGFR on breast cancer cell lines was evaluated using a rat monoclonal anti-EGFR antibody conjugated to FITC (Invitrogen, MA5-28104). To assess FAS expression, a CD95 (APO-1/Fas) monoclonal antibody (Invitrogen, 17–0959−42) was used. Flow cytometry analysis was conducted on a BD Accuri™ C6 Plus flow cytometer (BD Biosciences, San Jose, CA, USA), and the data was analyzed using FlowJo software (Ashland, OR, USA).

### Immunoblot analysis

Immunoblot analysis was performed to detect EGFR expression in breast cancer cell lines. The cells were lysed in RIPA buffer containing protease and phosphatase inhibitors, and the extracted proteins were separated by 12% sodium dodecyl sulfate‒polyacrylamide gel electrophoresis (SDS‒PAGE) before being transferred onto a nitrocellulose membrane. The membrane was blocked with 5% bovine serum albumin (BSA) in Tris-buffered saline with 0.1% Tween-20 (TBST) and subsequently probed with an EGFR monoclonal antibody (Invitrogen, MA5-13269) and a beta-actin mouse monoclonal antibody (Invitrogen, MA1-140). To evaluate FAS signaling protein expression, treated breast cancer cells were lysed, and their proteins were separated by SDS‒PAGE and probed with an anti-Ras (mutated G12D) antibody (Abcam, ab221163), a PI3K recombinant rabbit monoclonal antibody (Invitrogen, MA5–32070), and a beta-actin monoclonal antibody. Additionally, to assess the levels of apoptosis-related proteins, a cytochrome C monoclonal antibody (Invitrogen, 45–6100), a caspase 3 monoclonal antibody (Invitrogen, 43–7800), and a caspase 8 monoclonal antibody (Invitrogen, MA1–41280) were utilized. The membrane was then incubated with horseradish peroxidase (HRP)-conjugated secondary antibody (Invitrogen), and the immunoreaction was developed via SuperSignal™ chemiluminescent substrate (Thermo Fisher Scientific, Waltham, MA, USA). The signal was detected via a G: BOX chemiluminescence imaging system (Syngene) and quantified via ImageJ software (National Institutes of Health, Bethesda, MD, USA).

### Immunofluorescence staining

EGFR staining was performed on the following breast cancer cell lines. The cells were cultured on glass coverslips placed in a 24-well culture plate and subsequently fixed with 4% paraformaldehyde for 10 min. Permeabilization was achieved via the addition of 0.1% Triton X-100 in PBS for 10 min. To block nonspecific binding, the cells were incubated with 10% normal goat serum in PBS for 1 h. The cells were then treated with a mouse anti-EGFR monoclonal antibody (1:100 dilution, Invitrogen, MA5-13269) overnight at 4 °C, followed by a 1-hour incubation with Alexa Fluor 488-conjugated goat anti-mouse IgG (1:500 dilution) at room temperature. Nuclear staining was performed with Hoechst 33,342 (1 µg/ml; Thermo Fisher Scientific) for 10 min. Coverslips were mounted onto glass slides using ProLong Gold antifade reagent (Invitrogen, USA). Immunofluorescence images were acquired via a Ti-S Intensilight Ri1 NIS-D inverted fluorescence microscope (Nikon, Tokyo, Japan) at 40× magnification.

### Cell proliferation and cell viability assays

The proliferation or viability of breast cancer cell lines in response to EGF alone or in combination with CBD was assessed via the PrestoBlue™ Cell Viability Reagent (Invitrogen, Auckland, New Zealand) following the manufacturer’s instructions. The cells were seeded into 96-well plates and allowed to adhere for 24 h. Various concentrations of EGF (Sigma‒Aldrich, Darmstadt, Germany), ranging from 12.5 to 160 ng/mL, were then applied for 24 h. For the viability assay, the cells were treated with 5 ng/mL EGF and 20 µM CBD for 24 h. Following treatment, PrestoBlue™ reagent was added, and the cells were incubated at 37 °C for 1 h. The absorbance was measured at excitation and emission wavelengths of 560 nm and 590 nm, respectively, with a control wavelength for normalization. Relative fluorescence unit (RFU) values were obtained and analyzed using BioTek Gen5™ software (Agilent, CA, USA). The percentages of surviving cells were calculated by comparing the viability of the treated cells to that of the untreated controls, which were set as 100%.

### NK-92 cell killing assay

A coculture assay was performed to evaluate the effect of CBD pretreatment on the susceptibility of breast cancer cells to NK-92 cell-mediated cytotoxicity. NK-92 cells were cocultured with MDA-MB-468 or MDA-MB-231 cells at an effector-to-target (E: T) ratio of 2:1 for 24 h. The culture medium was supplemented with 5 ng/mL of EGF to stimulate FAS expression in the breast cancer cells, with or without the addition of 20 µM CBD. After co-culture, adherent cells—primarily TNBC cells—were separated from non-adherent NK-92 cells by carefully removing the supernatant and gently washing the wells. The viability of the adherent cell population was then assessed using the PrestoBlue™ Cell Viability Reagent, and immunoblot analyses were performed exclusively on this fraction to ensure that the results accurately reflected changes in TNBC cells. Cell viability was expressed as a percentage relative to untreated controls, which were defined as 100%.

### Statistical analysis

All experiments were conducted in triplicate, and the data are presented as means ± standard deviation (SD). Prior to statistical analysis, the homogeneity of variances was evaluated using the Brown–Forsythe test, which is well-suited for small sample sizes and non-normal distributions. For comparisons involving three or more groups within each cell line, one-way analysis of variance (ANOVA) was used to assess treatment effects. Tukey’s Honestly Significant Difference (HSD) test was applied for all pairwise comparisons. In dose–response analyses in which multiple EGF concentrations were compared to a single control (0 ng/mL), Dunnett’s post hoc test was performed following one-way ANOVA. For comparisons between two groups, independent-samples t-tests were conducted. A p-value of less than 0.05 was considered statistically significant. All statistical analyses were performed using GraphPad Prism version 7.0 (GraphPad Software, CA, USA).

## Results

### EGFR expression in triple negative breast cancer (TNBC) cell lines

**Fig. 1:**
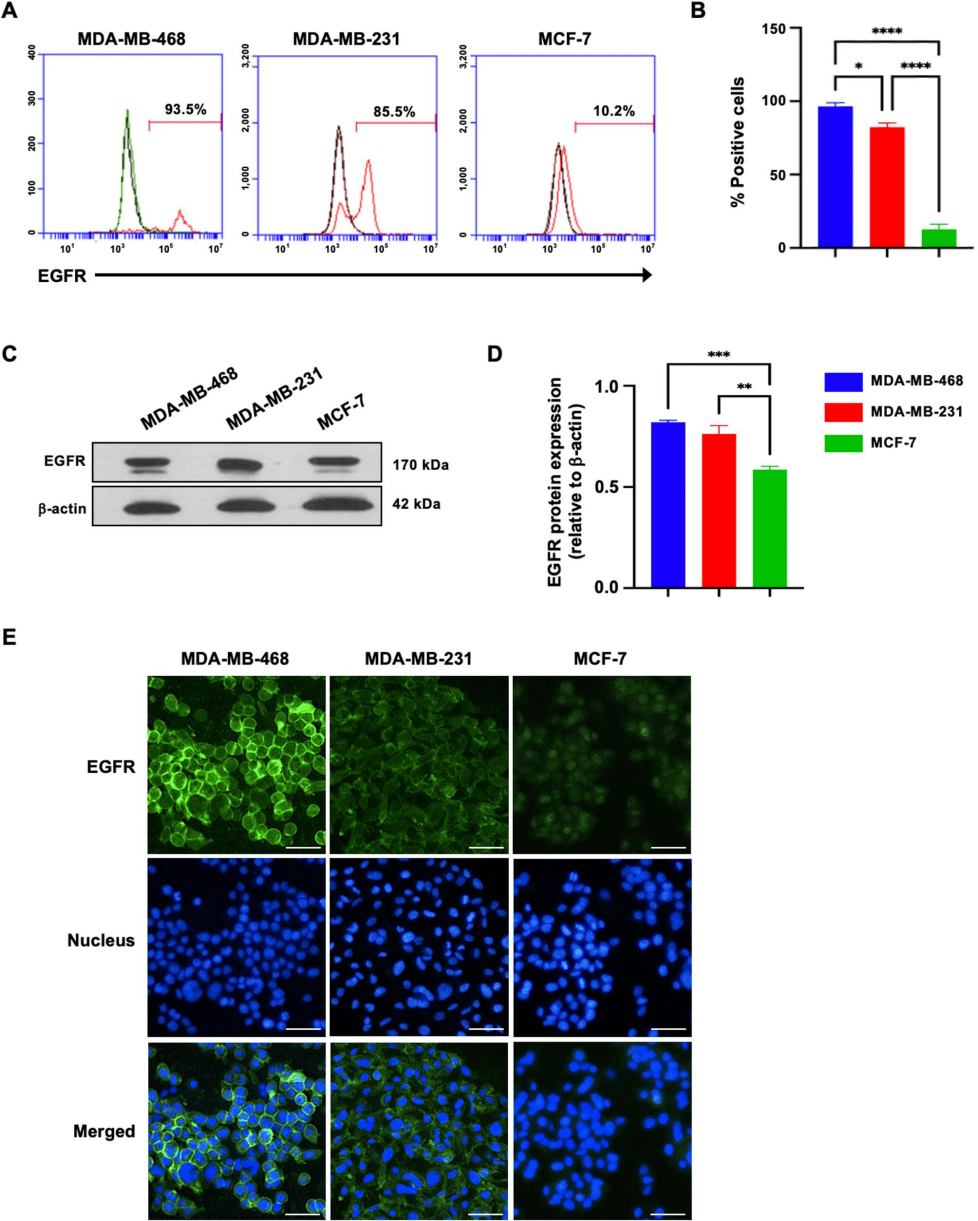
EGFR expression in TNBC cell lines compared with that in non-TNBC cell lines. **A** Flow cytometry histogram showing EGFR expression (red) alongside the corresponding isotype control (black). **B** Quantification of the proportion of EGFR-positive cells from three independent experiments. **C** Immunoblot analysis of EGFR and β-actin. **D** Densitometric quantification of the EGFR protein level relative to that of β-actin. **E** Immunofluorescence staining using an anti-EGFR monoclonal antibody (green) in breast cancer cell lines, with nuclei counterstained with Hoechst 33342 (blue). The scale bars represent 100 μm. The results are presented as the means ± standard deviation (SD). Statistical significance was evaluated using one-way Analysis of Variance (ANOVA) followed by Tukey’s Honestly Significant Difference (HSD) post-hoc test, with significance levels indicated as * *p* < 0.05, ** *p* < 0.01, *** *p* < 0.001, and **** *p* < 0.0001

In previous studies using immunohistochemistry (IHC), EGFR was found to be overexpressed in a subgroup of triple-negative breast cancer (TNBC) patients [5]. To confirm EGFR expression in TNBC cell lines, we used more sensitive and quantitative methods, including flow cytometry, immunoblotting, and immunofluorescence analysis (IFA), on TNBC cell lines (MDA-MB-468 and MDA-MB-231) and a non-TNBC cell line (MCF-7). Flow cytometry (Fig. 1A, B**)** revealed a significantly higher proportion of EGFR-positive MDA-MB-468 (96.40 ± 2.52%) and MDA-MB-231 (82.25 ± 2.91%) cells than MCF-7 (12.64 ± 3.51%) cells (*p* < 0.0001) (Fig. 1B). Consistent with these findings, immunoblot analysis revealed higher EGFR protein levels in MDA-MB-468 and MDA-MB-231 cells (0.82 ± 0.01 and 0.76 ± 0.04 relative to β-actin, respectively) than in MCF-7 cells (0.59 ± 0.02) (*p* < 0.01 and *p* < 0.001) (Fig. 1C, D). Additionally, IFA confirmed these results, revealing a significantly greater proportion of EGFR-positive cells and stronger EGFR staining intensity in TNBC cell lines than in non-TNBC cell lines (Fig. 1E). These results highlight the significantly elevated EGFR expression in TNBC cell lines, suggesting that EGFR plays a critical role in TNBC biology and may serve as a potential therapeutic target for TNBC patients.

### Effects of EGF on cell proliferation and FAS expression in TNBC cells

**Fig. 2:**
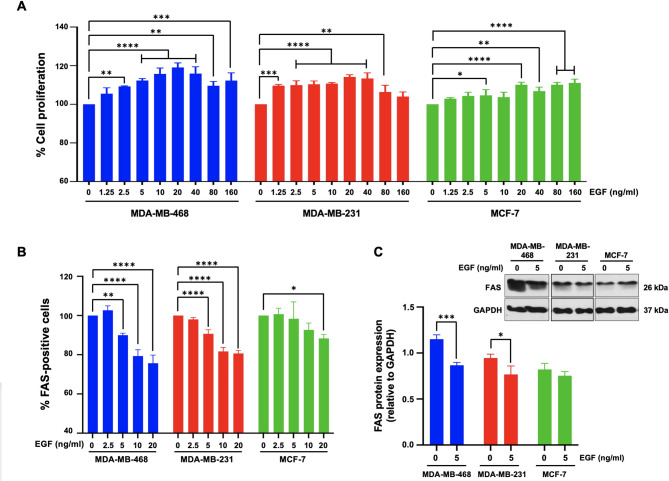
Effects of EGF on cell proliferation and FAS expression in TNBC cells. **A** TNBC and non-TNBC cell lines were treated with varying concentrations of EGF for 24 h, after which cell proliferation was assessed. **B** Flow cytometry analysis was performed to quantify the proportion of FAS-positive cells following 24 h of EGF treatment. **C** Immunoblot analysis was used to evaluate the expression levels of FAS and GAPDH, with densitometric quantification of FAS normalized to GAPDH. The data represent the results of at least three independent experiments and are expressed as the mean ± standard deviation (SD) (*N* = 3). For panels (**A**) and (**B**), statistical significance was determined by One-way ANOVA followed by Dunnett’s multiple comparison test, comparing each EGF concentration to the 0 ng/ml EGF control within each cell line. For panel (**C**), statistical significance was determined using an independent samples t-test comparing 0 ng/ml EGF to 5 ng/ml EGF within each cell line. * *p* < 0.05, ** *p* < 0.01, *** *p* < 0.001, **** *p* < 0.0001

Epidermal growth factor (EGF) is a potent mitogen that binds to the epidermal growth factor receptor (EGFR), activating downstream signaling pathways that promote cell proliferation and survival [29]. To assess the effects of EGF on breast cancer cell growth, we employed the PrestoBlue™ Cell Viability Assay, which specifically focuses on TNBC cell proliferation. The cells were treated with EGF at concentrations ranging from 12.5 to 160 ng/mL for 24 h, after which cell proliferation was measured. As shown in Fig. 2A, EGF treatment resulted in a dose-dependent increase in cell proliferation across all three cell lines. Notably, compared to non-TNBC MCF-7 cells, MDA-MB-468 and MDA-MB-231 cells presented significantly higher proliferation rates in response to EGF.

We further examined the impact of EGF on the expression of FAS (CD95/APO-1), a death receptor regulated by EGF, via flow cytometry. Figure 2B shows that EGF treatment significantly reduced the proportion of FAS-positive cells across all three cell lines. Compared to those in untreated control cells, FAS-positive cell populations in MDA-MB-468 cells were markedly decreased at EGF concentrations of 5, 10, and 20 ng/mL (90.0 ± 1.0%, 79.3 ± 3.2%, and 75.7 ± 4.1%, respectively) (*p* < 0.05). Similarly, in MDA-MB-231 cells, FAS expression significantly decreased at EGF concentrations of 5, 10, and 20 ng/mL (90.6 ± 2.1%, 81.7 ± 2.1%, and 80.7 ± 1.6%, respectively) compared with that in untreated controls (*p* < 0.05). In contrast, MCF-7 cells exhibited a significant reduction in FAS expression (88.3 ± 2.1%) only at the highest EGF concentration of 20 ng/mL (*p* < 0.05) (Fig. 2B). These results suggest that EGF downregulates FAS in TNBC cells, potentially decreasing their susceptibility to immune-mediated apoptosis. Immunoblot analysis via an anti-FAS antibody confirmed the downregulation of FAS protein (26 kDa) in EGF-treated MDA-MB-468 and MDA-MB-231 cells compared with that in untreated controls (Fig. 2C). However, no significant changes in FAS expression were observed in MCF-7 cells.

### Effects of cannabidiol on EGF-induced cell proliferation and the FAS signaling pathway

To assess the toxicity of cannabidiol (CBD), MDA-MB-468, MDA-MB-231, and MCF-7 cells were exposed to increasing concentrations of CBD (0–320 µg/ml) for 24 h. Using the PrestoBlue™ Cell Viability Assay, a dose-dependent decrease in cell viability was observed at higher concentrations of CBD. The CC_50_ values were 36 µg/mL for MDA-MB-468 cells, 35 µg/mL for MDA-MB-231 cells, and 53 µg/mL for MCF-7 cells (Supplementary Fig. 1). A concentration of 20 µM was selected as the nontoxic concentration for subsequent experiments in all three cell lines. Future research is needed to clarify the mechanisms underlying the differential cytotoxicity of CBD and explore its potential as a safe and effective therapeutic agent for breast cancer.

**Fig. 3:**
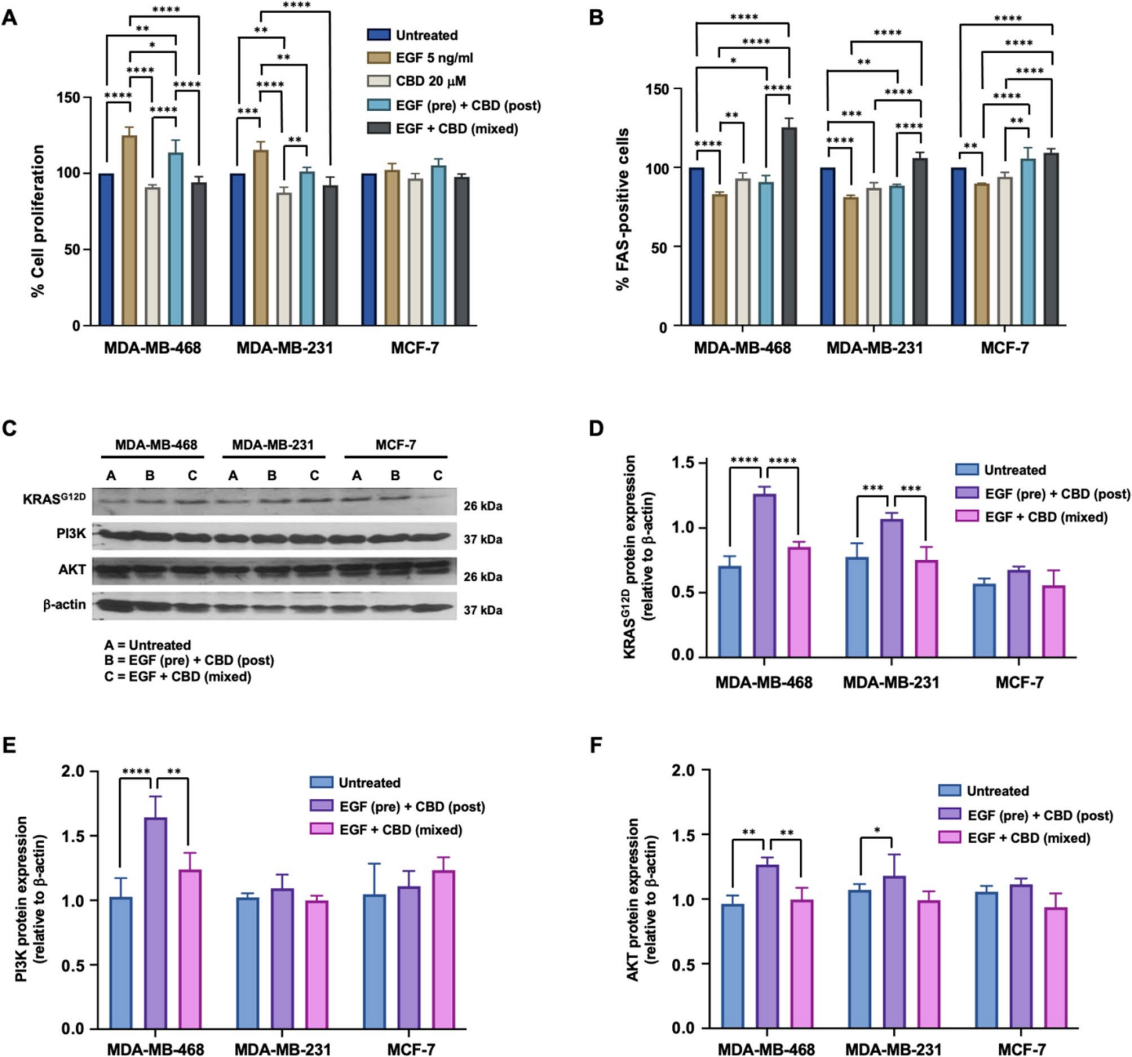
Effects of cannabidiol (CBD) on EGF-induced cancer cell growth and signaling. Breast cancer cell lines were treated with EGF and CBD for 48 h. **A** Cell proliferation was measured via the PrestoBlue™ Cell Viability Assay, with the untreated group exhibiting 100% proliferation. **B** The percentage of FAS-positive cells was determined by flow cytometry. **C** Immunoblot analysis was used to assess the expression of KRAS, PI3K, AKT, and β-actin. Densitometric analysis was performed for (**D**) KRAS, **E** PI3K, and **F** AKT, which were normalized to β-actin. The data represent the means ± standard deviations (SDs) from at least three independent experiments (*N* = 3). Statistical significance was determined by one-way Analysis of Variance (ANOVA) with Tukey’s Honestly Significant Difference (HSD) post-hoc test, with significance indicated as * *p* < 0.05, ** *p* < 0.01, *** *p* < 0.001, and **** *p* < 0.0001

Previous studies have suggested that cannabidiol (CBD), a crucial immune checkpoint receptor in cancer cells, can regulate FAS expression [30, 31]. However, the precise mechanisms involved and their therapeutic relevance remain unclear. To address this gap, we investigated the impact of CBD on FAS expression and KRAS signaling in TNBC cell lines, which frequently harbor KRAS mutations and exhibit EGF/EGFR-driven KRAS activation [32, 33]. MDA-MB-468, MDA-MB-231, and MCF-7 cells were subjected to various treatments, including EGF alone, CBD alone, EGF followed by CBD (pretreatment), or a combination of EGF and CBD (mixed), for 48 h. Compared to that in untreated cells, cell proliferation analysis (Fig. 3A) revealed a significant increase in the number of MDA-MB-468 (125.29 ± 5.87%) and MDA-MB-231 cells (105.91 ± 3.55%) following EGF treatment (*p* < 0.0001). In contrast, compared with no treatment, CBD alone significantly decreased the proliferation of MDA-MB-231 cells (87.38 ± 3.52%, *p* < 0.001). Pretreatment with CBD slightly increased proliferation only in MDA-MB-468 cells (113.66 ± 8.16%, *p* < 0.001). However, when EGF and CBD were applied simultaneously, cell proliferation significantly decreased in both the MDA-MB-468 and MDA-MB-231 cells (94.11 ± 3.80% and 92.19 ± 5.33%, respectively) compared with that in the EGF-only group (Fig. 3A). Notably, MCF-7 cells showed no significant changes across all treatment conditions. These results indicate that while EGF significantly enhances cell proliferation in all cell lines (*p* < 0.0001), CBD has an inhibitory effect, particularly in TNBC cells, and the combined treatment reversed EGF-induced proliferation.

FAS expression was significantly higher in the mixed treatment group than in the EGF alone group in the MDA-MB-468 cells (125.29 ± 5.87% vs. 83.07 ± 1.30%, *p* < 0.0001). Similar trends were observed in MDA-MB-231 (105.91 ± 3.55% vs. 81.17 ± 1.16%, *p* < 0.0001) and MCF-7 cells (109.21 ± 2.64% vs. 89.93 ± 0.15%, *p* < 0.0001) (Fig. 3B).

To further investigate the proteins involved in the EGF/FAS signaling pathway, we conducted an immunoblot analysis of KRAS (24 kDa), PI3K (86 kDa), and AKT (56 kDa) under various conditions (Fig. 3C)**.** The KRAS protein levels significantly increased in both TNBC cell lines following EGF pretreatment but decreased notably in the mixed treatment group (*p* < 0.0001 for MDA-MB-468 and *p* < 0.001 for MDA-MB-231) (Fig. 3D). PI3K expression significantly increased in MDA-MB-468 cells following EGF pretreatment (*p* < 0.0001), while it decreased in the mixed treatment group (*p* < 0.01). However, no significant changes were observed in the MDA-MB-231 and MCF-7 cells (Fig. 3E). Similarly, AKT expression significantly increased in both TNBC cell lines following EGF pretreatment (*p* < 0.01 in MDA-MB-468 cells and *p* < 0.05 in MDA-MB-231 cells) but was notably reduced in the mixed treatment group, specifically in MDA-MB-468 cells (*p* < 0.01) (Fig. 3F). These results indicate that mixed treatment significantly downregulates the expression of KRAS, PI3K, and AKT in both TNBC cell lines. Compared to β-actin (42 kDa), EGF pretreatment followed by CBD significantly increased KRAS in MDA-MB-468 cells (*p* < 0.0001) but downregulated it in the mixed treatment group (*p* < 0.0001). Similar patterns were observed for PI3K (*p* < 0.0001, *p* < 0.01) and AKT (*p* < 0.01, *p* < 0.01). MDA-MB-231 cells showed a similar trend for KRAS (*p* < 0.001), although no significant changes were detected for PI3K or AKT (*p* > 0.05).

### CBD improves TNBC cell susceptibility to NK-92 cell-mediated cytotoxicity

Given that CBD upregulates FAS and suppresses EGF-induced KRAS signaling in TNBCs, we hypothesized that CBD may increase TNBC cell susceptibility to immune cell-mediated cytotoxicity. To test this hypothesis, we first evaluated the cytotoxic effects of CBD on immune cells. The half-maximal cytotoxic concentration (CC_50_) for human peripheral blood mononuclear cells (PBMCs) was determined to be 9.54 µM after 24 h and 7.35 µM after 48 h (unpublished data). In contrast, NK-92 cells exhibited greater tolerance to CBD, with CC_50_ values of 101.31 µM and 44.13 µM after 24 and 48 h, respectively (unpublished data). These results suggest that NK-92 cells are more resistant to CBD-induced cytotoxicity than are PBMCs. Notably, T and B lymphocytes constitute the major subpopulations of human PBMCs, whereas NK and NKT cells constitute the minor subpopulations.

**Fig. 4:**
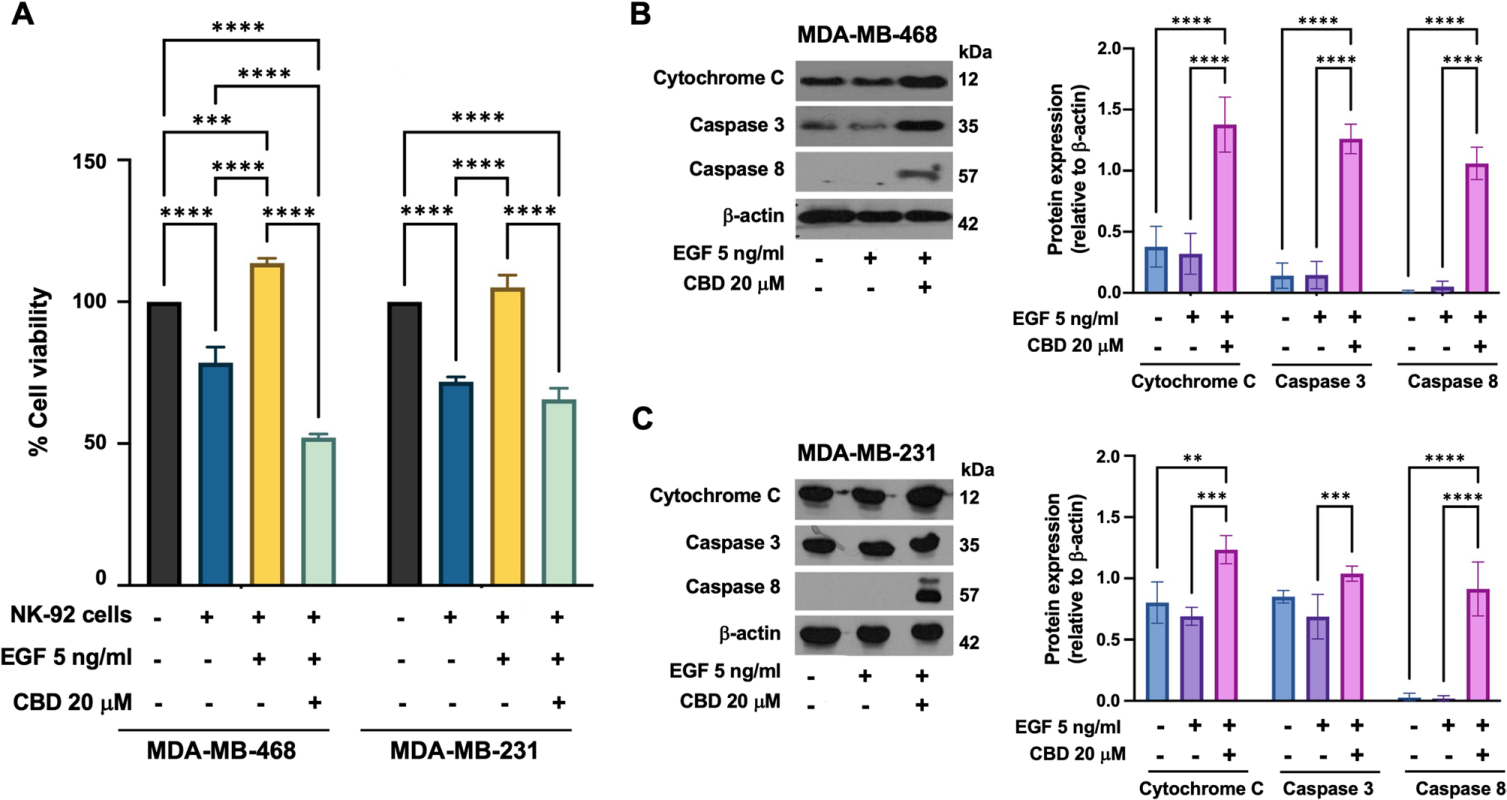
Effect of cannabidiol (CBD) on TNBC cell sensitivity to NK-92 cell-mediated cytotoxicity. TNBC cells were pretreated with either EGF alone or in combination with CBD before being cocultured with NK-92 cells at an effector-to-target (E: T) ratio of 2:1 for 24 h. **A** The percentage of viable cells following NK-92 cell-mediated cytotoxicity. **B** and (**C**) Immunoblot analysis showing the expression levels of cytochrome C, caspase-8, and caspase-3, normalized to those of β-actin, compared to those of untreated controls. Data were collected from at least three independent experiments, and the results are expressed as the means ± standard deviations (SDs) (*N* = 3). Statistical significance was determined by two-way analysis of variance (ANOVA) followed by Tukey’s post hoc test (*p < 0.05, **p < 0.01, ***P < 0.001, and ****P < 0.0001)

To investigate this potential enhancement of NK-92 cell-mediated cytotoxicity, TNBC cells were pretreated with EGF alone or a combination of EGF and CBD, followed by coculture with NK-92 cells at an effector-to-target ratio of 2:1 for 24 h. In the EGF-only condition, the viability of TNBC cell significantly increased compared to that of the untreated control, with MDA-MB-468 cells showing a viability of 113.69 ± 1.68% compared with 78.52 ± 5.49% (*p* < 0.0001) and MDA-MB-231 cells showing a viability of 105.07 ± 4.35% compared with 71.82 ± 4.35% (*p* < 0.0001)(Fig. 4A). Conversely, under CBD treatment, NK-92 cells significantly reduced the viability of TNBC cells in both MDA-MB-468 and MDA-MB-231 cells to 52.12 ± 1.28% and 65.59 ± 3.97%, respectively, compared with untreated controls (Fig. 4A).

To further investigate the underlying mechanisms of this enhanced cytotoxicity, we examined the expression of key apoptotic markers involved in the extrinsic apoptosis pathway, including cytochrome C, caspase-8, and caspase-3. Compared to other conditions, immunoblot analysis revealed significant upregulation of these apoptotic markers in TNBC cells pretreated with EGF/CBD (Fig. 4B-C). Notably, the expression of caspase-8, a critical initiator of the extrinsic apoptosis pathway, was markedly increased (*p* < 0.0001) in the EGF/CBD treatment group but was undetectable in the control group (Fig. 4B-C).

## Discussion

Triple-negative breast cancer (TNBC) remains a significant therapeutic challenge due to its aggressive nature [1–3] and the limited treatment options available [5, 34]. Our findings suggest that cannabidiol (CBD) may play a promising role in addressing this unmet clinical need. CBD exhibited anti-proliferative effects by downregulating the expression of EGFR and KRAS, two key oncogenic drivers in TNBC [10, 11, 35, 36], thereby disrupting critical signaling pathways. Additionally, CBD upregulated FAS, a death receptor, which sensitized TNBC cells to immune-mediated cytotoxicity [12, 37, 38]. This is particularly relevant given the crucial role of immune responses in cancer control. These results highlight the potential of CBD as a therapeutic agent for TNBC. However, further research is needed to elucidate the precise mechanisms of its action and to assess its efficacy in preclinical models and clinical trials. Combining CBD with targeted therapies or immunotherapies may offer synergistic benefits and improve treatment outcomes.

EGFR is frequently overexpressed in TNBC, and elevated levels of EGFR are associated with a poor prognosis and high rates of recurrence rates [5, 34]. Our study confirmed higher EGFR expression in TNBC cells than in non-TNBC cells, as demonstrated by multiple methods (Fig. 1). These findings support previous findings that TNBC typically has higher EGFR levels than other breast cancer subtypes do [39, 40]. However, EGFR expression in TNBC varies significantly, with many tumors displaying low or absent levels [5], which poses a challenge to broadly targeting EGFR, as a substantial portion of TNBC patients may not benefit from such treatments. While elevated EGFR levels are commonly linked to TNBC, other studies have reported improved survival in ER-positive/HER2-negative breast cancer patients with high EGFR expression [40, 41]. Additionally, TNBCs with high EGFR expression tend to have lower immune infiltration and reduced cytolytic activity [40], suggesting that targeting EGFR could diminish the effectiveness of immunotherapies that rely on a robust immune response. EGF treatment significantly stimulates the proliferation of TNBC cell lines [42].

Our study is the first to demonstrate that EGF treatment not only stimulates proliferation but also downregulates FAS expression in TNBC cells, while no such effect was observed in non-TNBC cells (Fig. 2). These findings suggest a potential reliance of TNBC on EGFR signaling for its aggressive behavior. The downregulation of FAS implies that EGF suppresses cell death mechanisms in TNBC, suggesting a dual mechanism by which EGF promotes TNBC aggressiveness through both enhanced proliferation and suppression of FAS-mediated apoptosis.

CBD has been shown to exhibit growth-inhibitory effects in breast cancer cell lines [43], with variations depending on concentration, mode of administration, and duration of exposure [44–46]. Our data indicate a dose-dependent cytotoxic effect of CBD on breast cancer cell lines. Although concentrations below 5 µg/mL resulted in minimal toxicity, higher concentrations significantly reduced cell viability across all the cell lines (Supplementary Fig. 1). Although CBD does not directly upregulate FAS expression, it has demonstrated neuroprotective effects through activation of the PI3K/AKT pathway [47]. On the other hand, CBD can inhibit EGFR palmitoylation, thereby reducing downstream signaling, particularly in KRAS-mutant contexts, by suppressing PI3K activation and decreasing MYC abundance [48]. Furthermore, CBD has been shown to inhibit migration, invasion, and epithelial‒mesenchymal transition (EMT) in non-small cell lung cancer (NSCLC) by suppressing the PI3K/AKT pathway [49]. However, there is no direct evidence from these studies that CBD specifically suppresses EGF-induced KRAS signaling through the PI3K/AKT pathway. Our findings provide strong evidence supporting the potential of CBD as a novel therapeutic strategy for TNBC. In particular, CBD treatment significantly increased FAS expression in TNBC cell lines (MDA-MB-468 and MDA-MB-231) (Fig. 3B). This upregulation of FAS may increase the susceptibility of TNBC cells to immune-mediated death, suggesting that CBD could be used to develop novel immunotherapeutic approaches.

KRAS is a well-established oncogene that is frequently implicated in the progression of TNBC [35]. Our data indicate that CBD suppresses EGF-induced activation of the KRAS signaling pathway in TNBC cells (Fig. 3D). Inhibition of this pathway may reduce tumor cell proliferation and survival [50, 51], contributing to the overall anticancer effects of CBD. Compared with non-TNBC cells, the selective cytotoxicity of CBD toward TNBC cells underscores its potential as a therapeutic agent. This differential sensitivity highlights the need for further investigation to clarify the underlying mechanisms and assess the potential of CBD as a targeted therapy specifically for TNBC. We observed significant differences between the sequential treatment condition (EGF pretreatment followed by CBD) and the simultaneous treatment condition (EGF combined with CBD). A plausible explanation for this discrepancy lies in the temporal dynamics of EGFR activation and its downstream signaling. EGF pretreatment may strongly activate EGFR-dependent proliferative and survival pathways, thereby reducing the effectiveness of subsequent CBD treatment. In contrast, simultaneous treatment (EGF + CBD) may enable CBD to interfere with or inhibit EGF-triggered signaling in real time, resulting in greater suppression of proliferation and enhanced induction of apoptosis, as evidenced by increased FAS expression. These findings underscore the significance of treatment timing and indicate that the interplay between EGFR signaling kinetics and CBD exposure plays a crucial role in determining therapeutic efficacy.

Natural killer (NK) cells are essential components of the innate immune system and play a critical role in tumor immunosurveillance. NK cell-based therapies [52, 53] are being investigated for various cancers, including TNBC, because of their ability to effectively target and eliminate cancer cells. Additionally, CBD has been shown to enhance the activation and effector memory differentiation of NKT-CIK cells, leading to increased antitumor activity [54]. The results of our coculture killing assay results (Fig. 4) provide strong evidence that CBD may synergize with NK cell therapy in the treatment of TNBC. Specifically, CBD treatment significantly increased the susceptibility of TNBC cells to NK-92 cell-mediated cytotoxicity. Previous studies have demonstrated that CBD enhances FAS expression in TNBC cells, potentially triggering extrinsic apoptosis upon interaction with its ligand [55]. This upregulation may sensitize TNBC cells to NK-mediated killing via the FAS pathway. Furthermore, our findings indicate that CBD treatment also upregulates the expression of caspase-8, a key initiator of apoptosis, in TNBC cells. These results suggest that CBD has the potential to augment the effectiveness of adoptive NK cell therapy for TNBC by promoting FAS-mediated apoptosis and activating caspase-8. Therefore, CBD could serve as a valuable adjuvant therapy to improve therapeutic outcomes in NK cell-based immunotherapies for TNBC. Although our findings strongly support the role of CBD in sensitizing TNBC cells to NK cell-mediated killing via EGFR and FAS modulation, we recognize that additional validation, including in vivo studies or experiments involving patient-derived TNBC cells, would further substantiate our observations. Future studies utilizing EGFR knockdown models or inhibitors might also clarify the precise mechanisms of interactions between CBD, EGFR signaling, and immune-mediated cytotoxicity, thereby strengthening the translational implications.

The therapeutic potential of CBD is promising; however, its cytotoxicity toward PBMCs highlights the need for careful administration [56, 57]. While CBD has anticancer properties, its long-term safety and efficacy remain incompletely understood [58–60]. Optimizing CBD dosing, exploring combinations with immunotherapies such as CAR-T-cell therapy [61], and employing targeted delivery strategies such as nanocarriers [62] are crucial steps in enhancing the therapeutic index. A comprehensive understanding of the effects of CBD on immune cells is essential for developing strategies that minimize adverse effects. In addition, investigating combination therapies [45, 63] with other anticancer agents may enhance therapeutic efficacy while reducing the toxicity associated with each individual drug.

## Conclusions

Our findings underscore the therapeutic potential of CBD in TNBC by targeting EGFR-driven pathways, modulating FAS expression, and enhancing immune-mediated killing. To fully realize the potential of CBD, further research is necessary to elucidate its complex mechanisms and assess its clinical efficacy in patients with TNBC. While CBD has significant anticancer properties, its cytotoxicity toward PBMCs necessitates careful dosing and administration strategies to minimize immune suppression. This study offers renewed hope for patients facing this challenging disease, positioning CBD as a potentially potent and multifaceted therapeutic agent.

## Supplementary Information

Supplementary Material 1.

## Data Availability

No datasets were generated or analysed during the current study.

## Abbreviations

CBD: Cannabidiol
EGF: Epidermal growth factor
EGFR: Epidermal growth factor receptor
FAS: Fas cell surface death receptor
TNBC: Triple-negative breast cancer
