# Supplementary material for: Cannabidiol sensitizes triple-negative breast cancer cells to NK cell-mediated killing via EGFR inhibition and FAS upregulation
Source: J Cannabis Res. 2025 Nov 4;7:85. doi: 10.1186/s42238-025-00340-5 (PMC12584368; doi:10.1186/s42238-025-00340-5)
Supplement: Supplementary file 1 — Supplementary Material 1. [file 42238_2025_340_MOESM1_ESM.pdf]

# Cannabidiol Sensitizes Triple-Negative Breast Cancer Cells to NK Cell-Mediated Killing via EGFR Inhibition and FAS Upregulation

Perawat Garunyapakun[1,2], Boonyanuch Ramwarungkura[3], Krissada Natungnuy[3,4], Chairat Turbpaiboon[1], Pa-Thai Yenchitsomanus[3,4], Mutita Junking[3,4,*]

[1] Department of Anatomy, Faculty of Medicine Siriraj Hospital, Mahidol University, Bangkok 10700, Thailand

[2] Anatomy Unit, Department of Medical Science, Faculty of Science, Rangsit University, Pathumthani 11110, Thailand

[3] Division of Molecular Medicine, Research Department, Faculty of Medicine Siriraj Hospital, Mahidol University, Bangkok 10700, Thailand

[4] Siriraj Center of Research Excellence for Cancer Immunotherapy (SiCORE-CIT), Faculty of Medicine Siriraj Hospital, Mahidol University, Bangkok 10700, Thailand

*Correspondence to: Mutita Junking,
Siriraj Center of Research Excellence for Cancer Immunotherapy (SiCORE-CIT) and Division of Molecular Medicine, Research Department, Faculty of Medicine Siriraj Hospital, Mahidol University, Bangkok 10700, Thailand
E-mail: mjunking@gmail.com

# Supplementary data

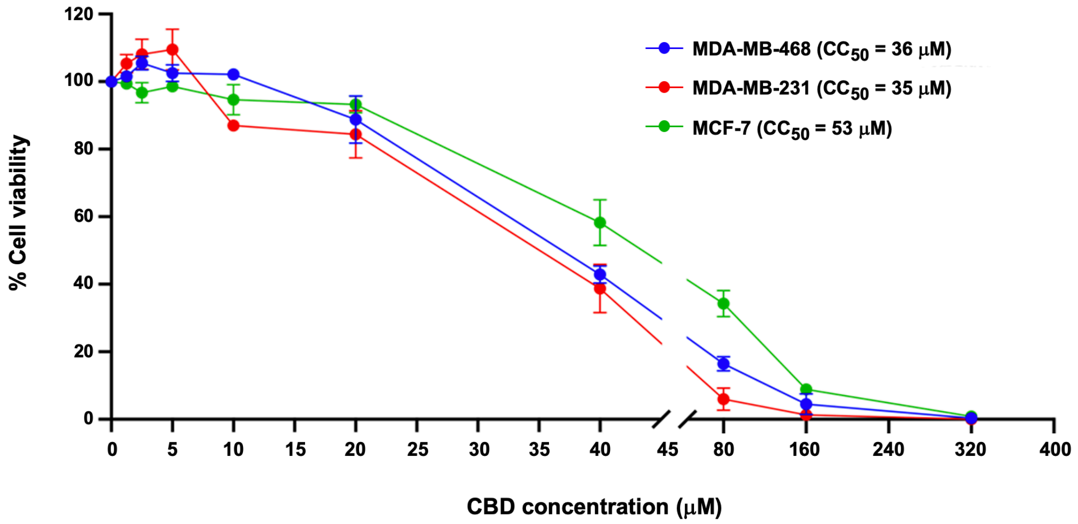


**Supplementary Figure 1. Cytotoxicity of CBD on breast cancer cell lines.** MDA-MB-468, MDA-MB-231, and MCF-7 cells were treated with CBD at the indicated concentrations for 24 hours. Cell viability was assessed using the PrestoBlue™ Cell Viability Assay. The cytotoxic concentration (CC50) was calculated to determine the concentration at which 50% of cells were affected by CBD treatment. Data are represented as mean ± SD (n=3 independent experiments).

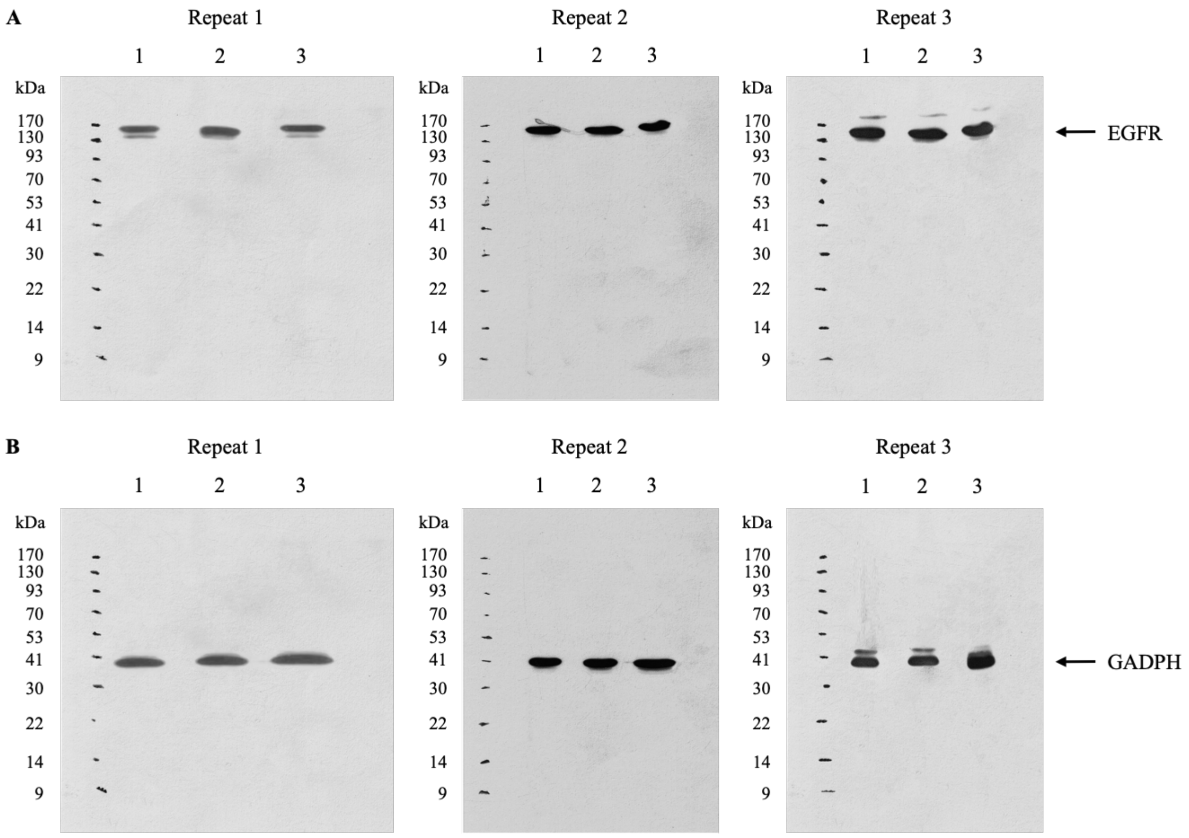


**Supplementary Figure 2. Immunoblot analysis of EGFR protein levels in breast cancer cell lines.** Immunoblot blot analysis was conducted to evaluate EGFR protein expression in three breast cancer cell lines: MDA-MB-468 (lane 1), MDA-MB-231 (lane 2), and MCF-7 (lane 3). Cell lysates were subjected to SDS-PAGE and transferred to nitrocellulose membranes. Immunoblotting was performed using anti-EGFR and anti-GAPDH antibodies, and chemiluminescence detection was performed using X-ray films. GAPDH served as a loading control.

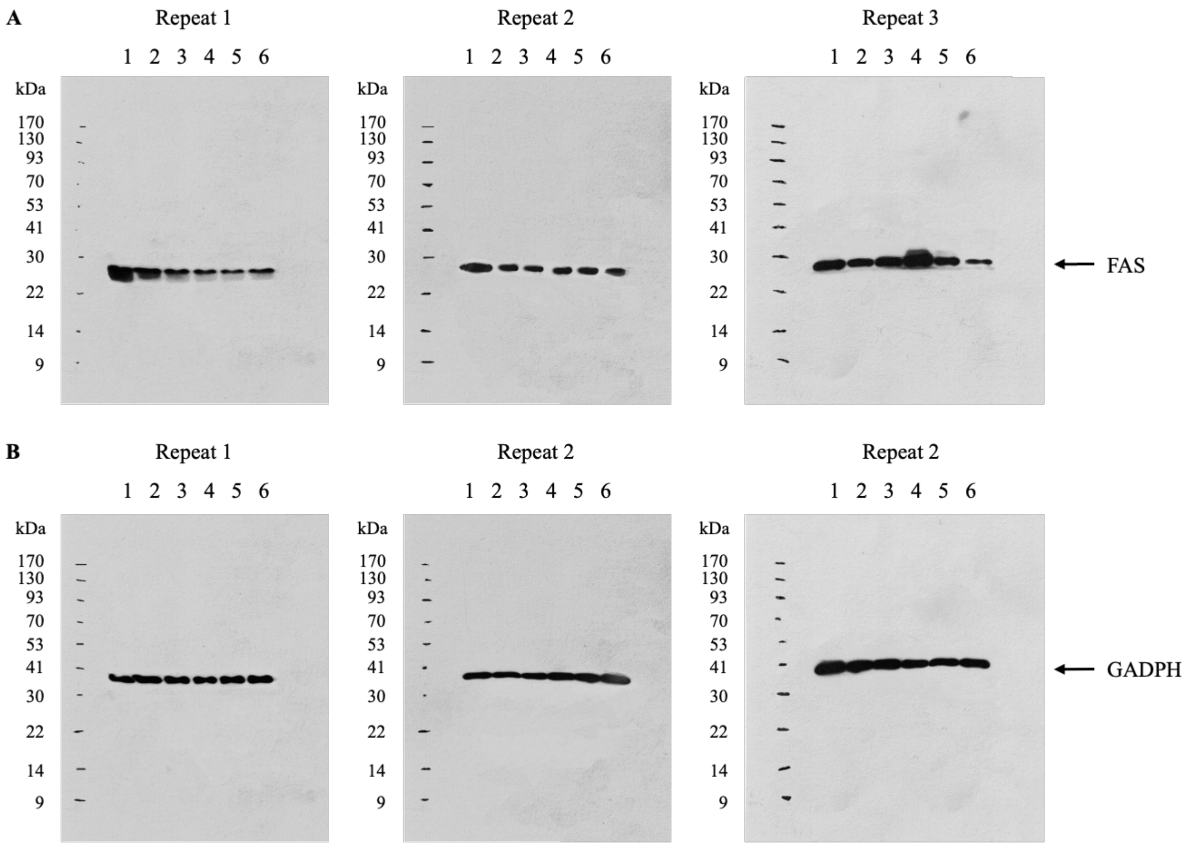


**Supplementary Figure 3**. **Immunoblot analysis of FAS protein levels in breast cancer cell lines.** Immunoblot blot analysis was conducted to assess FAS protein expression in three breast cancer cell lines without and with 5 µg/ml EGF. Cell lysates were subjected to SDS-PAGE and transferred to nitrocellulose membranes. Immunoblotting was performed using an anti-FAS antibody (A). GAPDH served as a loading control (B). Chemiluminescence detection was utilized with X-ray film for visualization. Lane 1: MDA-MB-468 untreated. Lane 2: MDA-MB-468 treated with EGF. Lane 3: MDA-MB-231 untreated. Lane 4: MDA-MB-231 treated EGF. Lane 5: MCF-7 untreated. Lane 6: MCF-7 treated with EGF.

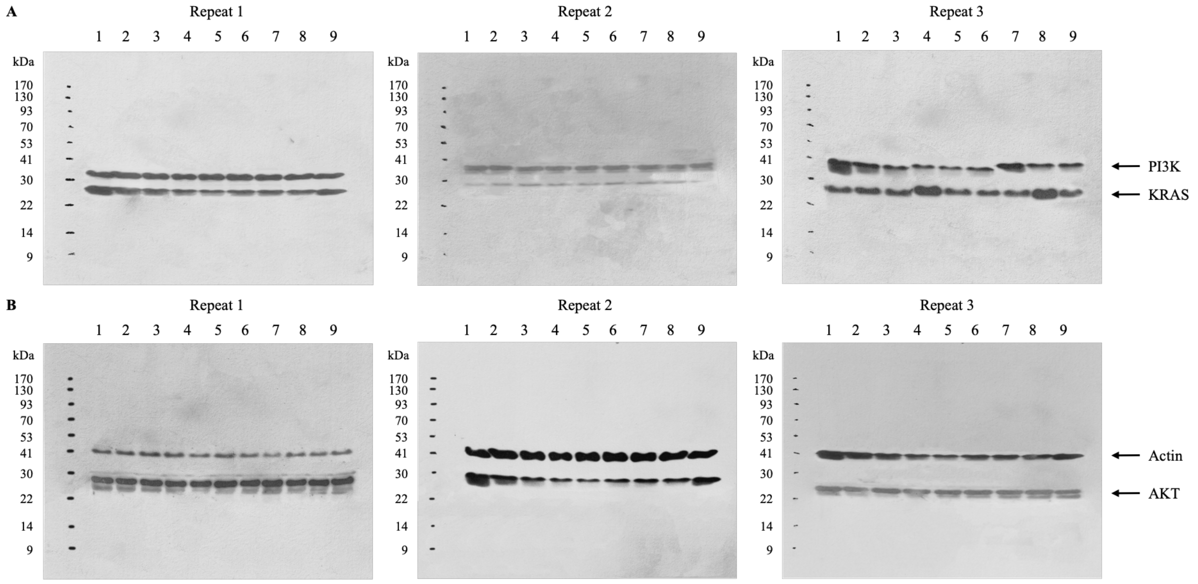


**Supplementary Figure 4. Effect of EGF and CBD on KRAS, PI3K, and AKT Signaling Proteins.** Western blot analysis was performed to assess the impact of epidermal growth factor (EGF) and cannabidiol (CBD) on the expression of KRAS, PI3K, and AKT signaling proteins in breast cancer cell lines. Cell lysates were subjected to SDS-PAGE and transferred to nitrocellulose membranes. Immunoblotting was conducted using antibodies against KRAS, PI3K, AKT, and β-actin, followed by chemiluminescence detection. Densitometric analysis was performed to quantify protein levels normalized to β-actin. A: Immunoblot analysis of KRAS and PI3K. B: Immunoblot analysis of AKT and β-actin. Experimental Conditions: Lane 1: MDA-MB-468 untreated, Lane 2: MDA-MB-468 pretreated with EGF following CBD, Lane 3: MDA-MB-468 treated with mixed EGF and CBD, Lane 4: MDA-MB-231 untreated, Lane 5: MDA-MB-231 pretreated with EGF following CBD, Lane 6: MDA-MB-231 treated with mixed EGF and CBD, Lane 7: MCF-7 untreated, Lane 8: MCF-7 pretreated with EGF following CBD, Lane 9: MCF-7 treated with mixed EGF and CBD.

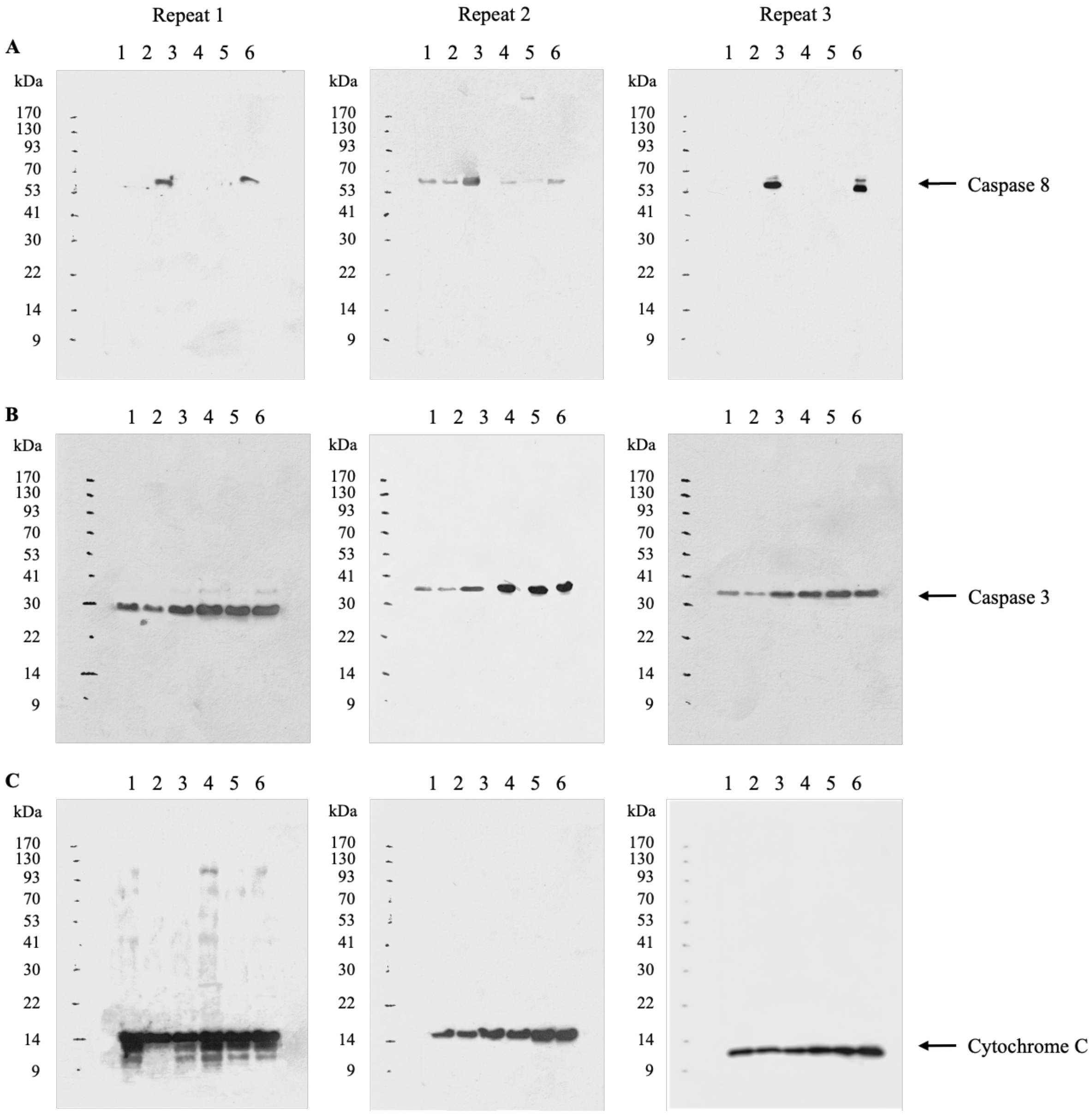


**Supplementary Figure 5. Effect of EGF and CBD on apoptosis protein markers.** Western blot analysis was conducted to assess the impact of epidermal growth factor (EGF) and cannabidiol (CBD) on apoptosis protein markers in breast cancer cell lines. Cell lysates were subjected to SDS-PAGE and transferred to nitrocellulose membranes. Immunoblotting was performed using antibodies against (A) caspase-8, (B) caspase-3, (C) cytochrome C, followed by chemiluminescence detection. Densitometric analysis was conducted to quantify protein levels normalized to β-actin. Data were obtained from at least three independent experiments. Lane 1: MDA-MB-468 untreated. Lane 2: MDA-MB-468 treated with EGF alone. Lane 3: MDA-MB-468 treated with mixed EGF and CBD. Lane 4: MDA-MB-231 untreated. Lane 5: MDA-MB-231 treated with EGF alone. Lane 6: MDA-MB-231 treated with mixed EGF and CBD.
